# Connexins: Synthesis, Post-Translational Modifications, and Trafficking in Health and Disease

**DOI:** 10.3390/ijms19051296

**Published:** 2018-04-26

**Authors:** Trond Aasen, Scott Johnstone, Laia Vidal-Brime, K. Sabrina Lynn, Michael Koval

**Affiliations:** 1 Translational Molecular Pathology, Vall d’Hebron Institute of Research (VHIR), Autonomous University of Barcelona, CIBERONC, 08035 Barcelona, Spain; laia.vidal.b@gmail.com; 2 Robert M. Berne Cardiovascular Research Center, University of Virginia School of Medicine, P.O. Box 801394, Charlottesville, VI 22908, USA; 3 Institute of Cardiovascular and Medical Sciences, College of Medical, Veterinary and Life Sciences, University of Glasgow, Glasgow G12 8TT, UK; 4 Division of Pulmonary, Allergy, Critical Care and Sleep Medicine, Department of Medicine, Emory University School of Medicine, Atlanta, GA 30322, USA; k.s.lynn@emory.edu; 5 Department of Cell Biology, Emory University School of Medicine, Atlanta, GA 30322, USA

**Keywords:** connexins, gap junctions, transcription, translation, post-translational modifications, trafficking

## Abstract

Connexins are tetraspan transmembrane proteins that form gap junctions and facilitate direct intercellular communication, a critical feature for the development, function, and homeostasis of tissues and organs. In addition, a growing number of gap junction-independent functions are being ascribed to these proteins. The connexin gene family is under extensive regulation at the transcriptional and post-transcriptional level, and undergoes numerous modifications at the protein level, including phosphorylation, which ultimately affects their trafficking, stability, and function. Here, we summarize these key regulatory events, with emphasis on how these affect connexin multifunctionality in health and disease.

## 1. Introduction

**Figure 1:**
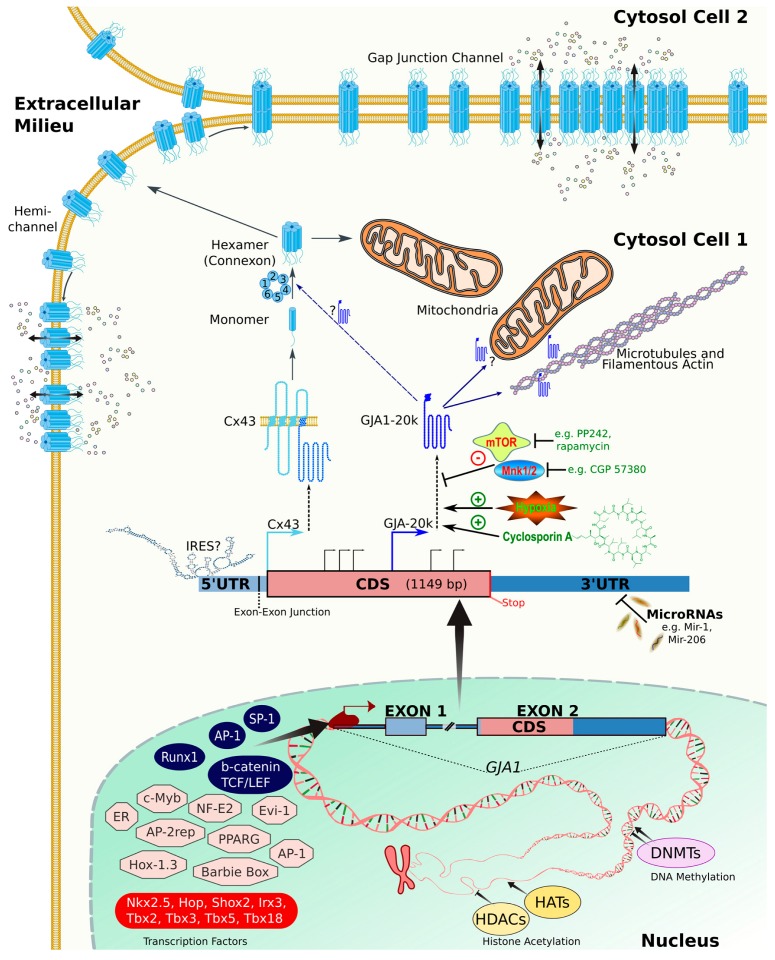
Connexins form hexameric connexons permeable to small molecules acting either as hemichannels or as intercellular channels. The human *GJA1* gene, encoding for connexin 43 (Cx43), contains two exons spanning a genomic region of 14,168 bp. Exon 1 contains 256 bp of the 5′ UTR (untranslated region), whereas exon 2 encompasses 16 bp of the 5′ UTR, the entire coding region (1149 bp), and the entire 3′ UTR region (1748 bp). Transcription of mRNA (3169 bp) is under regulation by numerous transcription factors as indicated in this figure and in the main text. Notably, Sp-1 and AP-1 are key regulators of Cx43 mRNA expression (grouped in blue). Multiple tissue-specific promoters are active, which has been well described in the heart (grouped in red). Additional transcription factors (grouped in light red) are derived from promoter analysis using the online Lasagna-Search tool (using a very strict cut-off of *p* < 0.0001 and Transfac transcription factor binding sites) [13]. Epigenetics regulate transcription, including through promoter hypermethylation by DNA methyltransferase enzymes (DNMTs). Acetylation by histone acetyltransferase enzymes (HATs) promote transcription, and the reverse reaction is mediated by histone deacetylases (HDACs). The transcript is also regulated by numerous microRNAs (see main text for details). In addition to full-length Cx43 (43 kilodalton (kDa)), the same mRNA can produce multiple truncated forms via internal translation initiation (indicated by arrows within the CDS (coding DNA sequence) of the mRNA, most notably the 20 kDa form named GJA1-20k). Truncated forms are also under translational regulation by a number of pathways such as mechanistic target of rapamycin (mTOR) and mitogen-activated protein kinase (MAPK)-interacting serine/threonine-protein kinase 1 (MNK1) and 2 (MNK2), and can be induced by inhibitors of these pathways as well as by other specific drugs such as cyclosporin A (the positive regulators are depicted in green). GJA1-20k is also induced by pathological states such as hypoxia. The function of GJA1-20k may include interaction with mitochondria and regulation of the actin cytoskeleton as well as regulation of Cx43 oligomerization and trafficking to the membrane. See main text for further details related to the figure.

Since the cloning of the first connexins in the 1980s, steady progress towards elucidating their regulation and function as signaling hubs and mediators of direct intercellular communication has been made [1,2,3]. All connexins share a conserved four-transmembrane domain structure that assembles into hexameric pores known as connexons that can integrate into the cell membrane (Figure 1). Hundreds to thousands of these connexons typically dock with opposing connexons in an adjacent cell, creating intercellular channels forming a clustered gap junction plaque that permits direct flux of ions and small cytosolic signaling molecules between cells, commonly referred to as gap junctional intercellular communication (GJIC) (Figure 1). More recently, connexons have been shown to act as “hemichannels” to facilitate direct exchange of molecules between the cell cytosol and the extracellular milieu under specific conditions [4]. Additionally, numerous noncanonical channel-independent functions have been described, in particular for connexin 43 (Cx43), which are mediated through direct protein interactions and modulation of signaling pathways [5]. The complexity and isoform-specificity of the connexin gene family is reflected by their links to numerous human diseases, many of which are rare syndromes with unique genotype–phenotype associations [6,7]. This latter phenomenon is underscored by the observation that mutations in different connexins can cause the same disease, whereas varying mutations in one connexin gene can result in vastly divergent diseases and phenotypes. Dysregulation of connexins is also increasingly linked to many common and often morbid medical conditions—such as stroke, heart attack, and cancer—which have been linked to the discovery of an expanding number of new functional attributes through both gap junction-dependent and -independent mechanisms [2,3,6,7,8,9]. As such, exploring the clinical and therapeutic potential of connexins as drug targets is pertinent and ongoing [10,11,12]. Towards this, a deeper understanding of how these genes and proteins are regulated and function is essential. This review aims to summarize and underscore important and unique mechanisms that regulate connexin function in healthy and diseased states, which ultimately shed light on clinical observations and future therapeutic opportunities.

## 2. Connexins: From Gene to Protein

### 2.1. Gene Structure and Splicing

Twenty-one human genes and 20 mouse genes encoding for connexin proteins have been identified, of which 19 are considered orthologous pairs [14,15]. The genes tend to have distinct chromosomal locations, although there are some regions of the genome containing clusters of connexin genes [14]. Most connexin genes share a common structure consisting of two exons separated by an intron of variable size. The majority of the 5′ UTR (untranslated region) is localized on exon 1, whereas the entire coding region and the 3′ UTR are found in exon 2. Some connexin genes contain more than two exons (for the 5′ UTR of the transcript), such as human *GJA5* (Cx40) [16], which contains three exons producing two distinct and tissue-specific transcripts, and *GJB6* (Cx30), described to contain six exons that allows for tissue-specific splicing [17]. Mouse connexin genes with three or more exons include *Gjb1* (Cx32) [18], *Gja1* (Cx43) [19], and *Gjc1* (Cx45) [20]. In a few cases, the coding region is also distributed over more than one exon [21,22,23,24]. A basal promoter (P1) is typically found within 300 bp upstream of the transcription initiation site of exon 1 [25]. However, splice isoforms have been reported due to alternate promoter usage, yielding different transcripts with the coding region being unaltered. As such, a deeper understanding of connexin gene structure, promoter usage, and splicing pattern is required for a full understanding of their impact in connexin-related diseases. For example, the human *GJB1* gene encoding Cx32 contains at least three exons (E1, E1B, and the coding exon E2) and produces two different alternatively spliced transcripts by using two tissue-specific promoters (P1 and P2) [26]. It is thus pertinent to include this region in mutational screening of dominant X-linked Charcot-Marie-Tooth (CMTX1) disease, a type of neuropathy that can be caused by mutations in Cx32 leading to defects in Schwann cell function, at least in cases where no mutations are found in the Cx32 coding region. Indeed, recent studies have identified mutations affecting *GJB1* splicing [27], and even deletion of the *GJB1* P2 promoter [28], as underlying causes of CMTX1. Others have shown that splicing mutations in *GJC2* encoding Cx47 can cause a severe form of Pelizaeus-Merzbacher-like disease [29]. Another splice-site mutation, in *GJB2* encoding Cx26, has been suggested to cause a mild postlingual onset form of hearing loss [30].

In addition to these more well-described biological phenomena, a few connexin pseudogenes (genes thought to originate from decay of genes that stems from duplication through evolution) have been identified in the human genome. The *GJA1* pseudogene (GJA1P) is located on human chromosome 5, whereas the regular *GJA1* gene encoding for Cx43 is located on chromosome 6. Although most pseudogenes are thought to be nonfunctional, GJA1P appears to be transcribed, possibly even translated, and may regulate tumor growth [31,32]. Mutations in GJA1P have also been associated with nonsyndromic deafness [33]. Functionally, GJA1P may influence *GJA1* expression levels by acting as a microRNA sponge [34]. In contrast, GJA6P seems to be a nonfunctional pseudogene, originated from the mouse *Gja6* connexin gene encoding Cx33, which has no human counterpart (Gene ID: 100126825). Another potential pseudogene has been inferred for *GJA4* (Gene ID: 100421028) encoding Cx37. The role of pseudogenes in disease is an emerging field, particularly among genes causing multiple different diseases or syndromic diseases, such as connexins.

### 2.2. Transcription Factors and Epigenetics

Connexins are expressed distinctively in almost all vertebrate cell types (excluding erythrocytes, mature sperm cells, and differentiated skeletal muscle cells) [35]. Some connexins (notably Cx43) are expressed in numerous cell types, whereas others show a more restricted expression profile (e.g., Cx50 that is mainly found in lens cells). Most tissues express multiple connexins. The epidermis of the skin, for example, is thought to express at least 10 different connexins whose expression partially overlap during keratinocyte stratification and differentiation [36,37,38]. Five of these connexins underlie 11 clinically different cutaneous disorders [37,39]. This spatiotemporal expression pattern is in large part controlled by transcription factors and epigenetic mechanisms. Several transcription factors acting as regulators of basal (ubiquitous) or cell-specific gene activity, and their upstream signal transduction pathways, have been implicated in the control of connexin expression (Figure 1). Notably, specificity protein 1 (Sp1), an important basal transcription factor that binds to GC box sequences in promoter regions, has been reported to favor transcriptional initiation of several connexin genes, including Cx26 [40], Cx32 [41,42], Cx40 [16,43,44,45,46,47], and Cx43 [45,48,49,50,51,52,53]. Examples of other important regulators that control connexin gene expression include: (i) Activator protein 1 (AP1) transcription factor, composed of proteins belonging to the c-Fos, c-Jun, activating transcription factor (ATF), and J domain containing protein( JDP) families that typically promote positive regulation. AP-1 sites have mainly been described in Cx43 [48,54,55], whereas putative sites have been identified in the Cx45 promoter [56]. (ii) The Wnt pathway: activation of this pathway leads to the formation of nuclear β catenin/TCF (T-cell factor) complexes that act as transcription factors by binding to specific TCF/LEF (lymphoid enhancer-binding factor) motifs present in the promoter of human *GJA1* and mouse *Gja1* encoding Cx43 [57]. From a physiological and disease point of view, this may also be relevant. For example, one study showed Wnt signaling could modulate Cx43-dependent GJIC in the heart, which ultimately may contribute to altered impulse propagation and arrhythmia in the myopathic heart [58]. The importance of GJIC in the heart is well documented and several cell-specific transcription factors have been shown to either activate or repress connexin gene expression in this setting (Figure 1 (red box), reviewed in [25,59]). These studies have revealed a role of: (i) homeobox proteins, transcription factors with a unique DNA-binding domain that target gene promoter sequences by self-complementarity (e.g., Nkx2.5, Hop, Shox2, Irx3); (ii) T-box (Tbx) proteins, transcription factors that possess a domain that recognizes a DNA binding element (e.g., Tbx2, Tbx3, Tbx5, Tbx18); and (iii) GATA proteins, important regulators of specific gene expression in different tissue (e.g., GATA-4) [25,59].

Besides the well-described transcriptional regulation of the cardiac connexins, other cases of tissue-specific regulation have been reported (for an overview, see [25]). Cx32 transcription has been found to be positively regulated by hepatocyte nuclear factor-1 (HNF-1) via Sp1 in liver cells [60], by the transcription factor Mist1 in secretory pancreatic acinar cells [61], and by the Sox10 in synergy with the early growth response-2 gene (Egr2) in Schwann cells [62]. This exemplifies how different transcription factors act in a tissue-dependent fashion. Complex transcriptional control thus allows for tissue-specific regulation of connexin expression. It also facilitates rapid response to environmental changes, for example, progesterone and estrogen act as positive and negative regulators, respectively, of Cx43 transcription in the myometrium during pregnancy and labor [63]. Transcription factors are also important during pathological states, such as in ischemia, where multiple connexins are emerging as important injury response mediators. Their roles in complex disease, such as cancer, are also being unraveled. In breast cancer, for example, Cx43 has been proposed to play a biphasic role acting both as a tumor promotor and a tumor suppressor depending on context such as cancer subtype and stage [3]. In this setting, the aforementioned role of progesterone and estrogen as regulators of Cx43 expression may be of importance. Recent evidence also suggests that the transcription factor FOXP3 directly binds to and inhibits RUNX1 in mammary epithelial cells, whereas in the absence of FOXP3 in breast tumors, RUNX1 downregulates Cx43 expression [64]. Understanding the role of transcription factors will provide further insight into loss and overexpression of connexins during tumor progression and other pathological states.

Connexin expression is also under significant epigenetic regulation (for recent extensive reviews see [25,65]). Two major epigenetic mechanisms have been described to regulate transcriptional control: DNA methylation and histone acetylation. Connexin gene inactivation due to hypermethylation of CpG islands in the promoter region has been described in various human carcinomas, including Cx26 in lung [66] and breast [67], Cx32 in a renal cell carcinoma cell line [68], and Cx43 in breast cancer [69]. In addition, a gradual decrease in Cx32 and Cx43 mRNA expression levels is associated with promoter hypermethylation in *Helicobacter pylori*-associated gastric tumorigenesis [70]. Transcriptional silencing via promoter hypermethylation is mediated by the enzyme DNA methyltransferase (DNMT). The use of demethylating drugs (DNMTs inhibitors), such as 5-aza-2-deoxycytidine and 5-azacytidine, has been proposed as a potential therapeutic solution in cancer, as an increase connexin expression and/or GJIC has been demonstrated in specific cases [68,71,72]. However, the correlation between hypermethylation and gene expression is not always direct and differs between connexin isoforms [72].

Histone acetylation and deacetylation—causing chromatin decondensation and condensation, respectively—constitute other important mechanisms of epigenetic regulation of connexin transcription [25,65]. While acetylation is catalyzed by histone acetyltransferase (HAT) enzymes and promotes transcription, the reverse reaction is mediated by histone deacetylase (HDAC) enzymes. Histone acetylation also affects connexin expression, and inhibitors of HDAC enzymes (HDACi)—such as trichostatin A, sodium butyrate, and 4-phenylbutarate—have been shown to enhance connexin and GJIC in a variety of cell populations, including in cancer cells [73], in which therapeutic and preventive roles for specific HDACi have been proposed. Histone deacetylase inhibition has also been shown to reduce Cx43 expression and gap junction communication in cardiac cells [74], which has implications with regards to potential side effects such as slow ventricular conduction or arrhythmias. Therefore, the action of HDACi seems to be connexin- and cell type-dependent. Curiously, Cx43 has been shown to influence histone acetylation of other genes; in a human pulmonary giant cell carcinoma cell line, the follistatin-like 1 (FSTL1) promoter was shown to be associated with acetylated histones H3 and H4 upon Cx43 transfection. Cx43 was proposed to act as a “histone deacetylase inhibitor” that modulates gene expression and inhibits tumor invasion [75].

The potential therapeutic role of epigenetic regulations has broad interest, particularly in complex diseases such as cancer, as exemplified above. However, the nonspecific nature of this gene regulatory mode complicates more direct and specific therapeutic targeting. Moreover, research is needed to determine if connexin levels are mainly mediated via HDACi histone modification [73,76], via non-histone protein modification of transcription factors, or via direct or indirect connexin protein modification such as Cx43 acetylation or phosphorylation [77,78,79].

### 2.3. RNA Stability and MicroRNAs

MicroRNAs (miRNAs) are short single-stranded noncoding RNAs that can regulate expression at a post-transcriptional level by base pairing to mRNA sequences (usually located at the 3′ UTR region), reducing protein expression levels via mRNA degradation, translational inhibition, or transient mRNA sequestration. Numerous microRNAs have been predicted to downregulate the expression of different connexin genes (for recent reviews see [65,80]). Cx43 is by far the best studied connexin, and a number of functional microRNAs targeting this gene have been identified, including miR-1, miR-23a, miR-186, miR-200a, miR-206, and miR-381 in human breast cancer [81], miR-20a in human prostate cancer [82], miR-221/222 in glioblastoma multiforme [83], and miR-206, miR-1, and miR-133 in cardiac myocytes and during skeletal myoblast differentiation [84,85,86].

Regulation of connexin expression by miRNAs has been described to be active in various disease states (for example, in cancer) by affecting hallmarks such as proliferation and invasion [82,83]. In therapeutic settings, options include targeting miRNAs that regulate connexins in order to reverse the malignant phenotype. This has been shown in several studies, including in human glioblastoma cells, where inhibition of miR-221/222 activity with antisense oligonucleotides led to the upregulation of Cx43 and restoration of GJIC [83].

As mentioned above, miR-1 acts in cardiac muscle and downregulates Cx43 expression. This has been related to several cardiopathologies in humans, including the regulation of cardiac arrhythmogenic potential [86]. In contrast, loss of miR-1, and thus increased Cx43 expression, has been linked to myotonic dystrophy [87]. Interestingly, a severe congenital heart defect, tetralogy of Fallot, is associated with downregulation of miR-1 and miR-206, which is thought to lead to an increase in Cx43 protein levels [88]. miR-1 downregulation of Cx43 in the bladder musculature has also been reported to have a role in overactive bladder syndrome [89].

Connexins are implicated in joint and bone disease [90]. Cx43 has an important role in osteoblast growth and differentiation, and various miRNAs (including miR-23a [91] and miR144-3p [92]) have been shown to target Cx43 in this setting. Cx43 can also influence the expression of miRNAs themselves, notably miR-21 in osteocytes, a pathway linked to osteocyte apoptosis and osteoclast formation/recruitment [93]. Moreover, direct transfer of miRNAs—through gap junctions—has been described, and is thought to play a role in bone development [94] as well as in aspects of tumor growth and tumor dormancy [3].

In addition to miRNAs, connexin transcript stability can be regulated by RNA-binding proteins (RBPs), such as human antigen R (HuR) that stabilizes the Cx43 mRNA by binding adenylate/uridine-rich elements (AREs) present in the Cx43 3′ UTR [95]. Other examples include S1516-binding protein elements, which may regulate Cx43 expression, particularly in Ras-transformed cancers [96]. For further insight into the epigenetic regulation of connexins, including by miRNAs, we refer to other more exhaustive recent reviews [65,80].

### 2.4. Translational Regulation

#### 2.4.1. Internal Ribosome Entry Site (IRES)

Due to the key role of connexins in sustaining many cellular functions and tissue physiology, it has been suggested that connexin expression needs to be maintained at all times, even under conditions where the classical cap-dependent mRNA translation pathway is suppressed, such as during mitosis, apoptosis, differentiation, senescence, or cell stress [97,98]. Several internal ribosome entry site (IRESs) elements have been reported in the mRNA of connexins, notably in Cx43 [99] (Figure 1), Cx32 [100], and Cx26 [101]. An IRES is a nucleotide sequence, usually located within the 5′ UTR of the mRNA, which—in contrast with the canonical translation mechanism—allows for cap-independent translation initiation, a process regulated by specific RBPs also known as IRES trans-acting factors (ITAFs) [102,103]. However, numerous other translation initiation mechanisms are thought to exist [104] and whether true IRES-mediated translation occurs in the aforementioned connexins and other family members, is subject to caution, and additional specific molecular assays are warranted [105]. Additional work is also needed towards elucidating their functional relevance. One study suggests that IRES-mediated translation of Cx26 and Cx43 occurs in density-inhibited cancer cells (where cap-dependent translation is reduced), thus leading to the induction of GJIC and potentially reduced tumor growth [101]. Some data also points towards an important role of IRES-translation of connexins in human physiology. Notably, a specific mutation in the 5′ UTR IRES sequence of Cx32 is linked to neurodegenerative Charcot–Marie–Tooth disease [100].

#### 2.4.2. Alternative Translation of Truncated Connexin Forms

Most IRES sequences are located in the 5′ UTR, yet a few examples exist (notably Notch2 [106]) where an IRES sequence is located within the coding region, allowing translation of truncated protein forms. A similar mechanism has been proposed for Cx55.5 in zebrafish, in which an 11 kilodalton (kDa) truncated C-terminal form is produced and localizes to the nucleus of outer retina cells [107,108].

In mammalian cells, the presence of truncated forms of Cx43 is often observed in immunoblots. In particular, a 20 kDa form (named GJA1-20k) is highly prevalent in cultured cells, which was described to arise from the Cx43 coding sequence and correspond to the C-terminal tail [109]. More recently, Smyth and Shaw described that GJA1-20k and several other less prevalent truncated forms can occur in normal tissue, and is due to internal translation initiation events [110]. Multiple groups have now confirmed this observation and further delineated key regulatory pathways, such as the mechanistic target of rapamycin (mTOR) [110,111] and the mitogen-activated protein kinase (MAPK)-interacting serine/threonine-protein kinase 1 (MNK1) and 2 (MNK2) [111] signaling cascades. Additionally, regulation occurs in response to important physiological conditions such as hypoxia [112] (Figure 1). Although an internal IRES element has been suggested [112], evidence suggests a highly unusual cap-dependent mechanism is critical for the efficient synthesis of these truncated forms [80,111].

The C-terminus of Cx43 has been extensively studied and is implicated in the regulation of a variety of biological events such as cell migration and proliferation, neuronal differentiation, and cytoskeletal changes (for a recent review see [5]). However, functional roles for specific internally truncated forms of Cx43 are currently being elucidated. Thus far, GJA1-20k has been shown to act as a potential chaperone for Cx43 [110,113] that facilitates microtubule-based mitochondrial transport and mitochondrial network integrity [114] (for details, see Section 4.3). Additionally, loss of GJA1-20k (but not full-length Cx43) has been reported in early-stage human breast cancers, followed by its re-expression in cell lines regulated by p53 activation via miR-125b [115]. Roles for these truncated forms of connexins in complex genetic disease is of future interest considering recent advancements in the potential for pharmacologic modulation of internal translation [80].

## 3. Post-Translational Regulation of Connexins

Post-translational modification of connexin proteins regulates many important aspects of their life-cycle, including synthesis, trafficking, channel gating, and protein–protein interactions. While highly conserved, variations occur throughout the connexin family in protein sequence, size of intracellular N-/C-terminus, and loop regions. Connexin extracellular loop regions contain disulfide bridges that form between cysteines to maintain membrane topology and facilitate docking with opposing connexons, allowing the formation of gap junctions [116]. Unlike many other membrane-bound proteins, connexins are not glycosylated, with membrane trafficking and protein folding being regulated through alternative pathways (for details, see Section 4). The relatively unstructured nature of intracellular connexin domains makes for an ideal environment for post-translational modification to induce conformational changes that regulate protein–protein interactions. The majority of connexins contain multiple consensus sites for modifications through phosphorylation, *S*-nitrosylation, SUMOylation, and others. There have been several recent and comprehensive reviews on connexin post-translational modifications [5,117,118,119]. Instead of recapitulating these articles, we will highlight some of the main aspects of post-translational modifications of connexins and discuss their relevance in human disease.

### 3.1. Phosphorylation

Phosphorylation is a key regulator of connexin proteins, hemichannels, and gap junction channels [120,121,122]. The addition of phosphate groups to specific amino acids—including serine (Ser, S), threonine (Thr, T), or tyrosine (Tyr, Y)—leads to changes in charge, hydrophobicity, and potential alterations in protein structure resulting from formation of hydrogen bond networks [123]. These can alter the way the connexin protein interacts with itself (e.g., channel regulation) or with other proteins (e.g., trafficking and protein–protein interactions).

Phosphorylation has been reported in a large number of connexins, e.g., Cx31 [124], Cx32 [125,126,127], Cx37, Cx40 and Cx45 [128,129], Cx43 [130,131], Cx46 and Cx50 [132,133,134], and Cx47 [135]. The majority of phosphorylation events are reported within the connexin C-terminus, with the exception of Cx26, which is not phosphorylated in its short 11 a.a. C-terminus [136,137]. However, mass spectrometry has demonstrated multiple potential Cx26 phosphorylation sites in the N-terminus, which are differentially regulated by hydroxylation, and further putative sites in the cytoplasmic loop, although the functions of these Cx26 phosphorylation sites are unknown [138,139]. There are some reports of intracellular loop phosphorylation—such as Cx56 [140] and Cx35 [141]—although this does not appear to be the case for Cx43 or other connexins [5,142,143]. There are no reports of N-terminus phosphorylation in other connexins, although Cx43–Ser5 is a potential candidate site [144]. The C-terminus of connexins are intrinsically disordered protein (IDP) regions with a high Ser/Thr/Tyr content, as described for Cx32, Cx40, Cx43, Cx45, and Cx50 [145,146,147,148,149]. Stable α-helical regions have been identified by nuclear magnetic resonance (NMR) and circular dichroism (CD) in the C-terminus of Cx43 [146,150] and other connexins, for example, Cx37, Cx45, and Cx50 [151,152,153,154]. However, stable alpha-helices are not a common feature of the connexin C-terminus. For instance, Cx40 only forms dynamic alpha-helices between Cys267–Gly285 [155]. Several lines of evidence—such as electrophoretic shifts on SDS-PAGE gels and NMR analysis—suggest that phosphorylation by enzymes, such as MAPK and Protein Kinase C (PKC), result in differential, transient increases in connexin C-terminal alpha-helical content [128,149,156,157,158].

The significance of the formation of alpha-helical domains is the potential for higher order secondary structures that regulate channel gating and protein partner binding. In Cx43, it has been demonstrated that the C-terminus interacts with the intercellular loop to regulate channel functions in a “ball-and-chain” type mechanism [159,160], although other factors relating to phosphorylation (e.g., charge and hydrophobicity) may also influence channel gating. Multisite phosphorylation of proteins is known to alter protein half-life, docking, and intracellular localization, which may also influence gap junction signaling [148,149,161]. Connexin 43, the most widely studied of the connexin family, has 30 putative phosphorylation sites which have been extensively demonstrated to be post-translationally modified, leading to alterations in gap junction signaling. For detailed reviews of these phosphorylation sites and their effects on channel regulation see [5,143,144,162,163,164]. The effects of post-translational modifications on connexins are also shown in Table 1.

Phosphorylation is a key regulator of physiological states in tissues, and changes in the phosphorylation status has been observed in several disease states. Within the vasculature, heterocellular endothelial cell–smooth muscle cell contacts, called the myoendothelial junctions (MEJs), express Cx37, Cx40, and Cx43, allowing for the direct exchange of intercellular signaling ions and molecules such as Ca^2+^ and IP_3_ [203,204]. At the MEJ, Cx43 and Cx37 are regulated by post-translational modifications, including phosphorylation and *S*-nitrosylation (for details, see Section 3.2). In vitro and ex vivo data demonstrate that gap junctions at MEJs allow for the movement of Ca^2+^ and IP_3_ between endothelial and vascular smooth muscle cells, which is in part regulated via Cx43-Ser368 [205].

In the healthy heart, Cx43 is primarily localized to the intercalated disc region of cardiomyocytes. Opening of Cx43-containing channels and signal conduction is facilitated by phosphorylation at residues including Ser365, 325, 328, and 330 [206,207,208]. Phosphorylation acts as a molecular switch, regulating gap junction opening. In ventricular arrhythmias following myocardial infarction, raised intracellular Ca^2+^ concentration leads to de-phosphorylation of Cx43-Ser365, which acts as the gatekeeper to phosphorylation of Cx43-Ser368. This resulting increase in Cx43-Ser368 reduces GJIC and promotes a redistribution of Cx43 to lateral regions of the cardiac myocytes, disrupting signaling in the heart [208,209,210].

Formation of large cardiac gap junction plaques at the intercalated disc is modulated through Cx43 interactions with zonula occludens 1 (ZO-1) [211,212,213,214]. In turn, this protein–protein interaction is regulated by PKC phosphorylation of Cx43 at Ser368, which inhibits ZO-1-mediated disassembly of gap junctions [215]. In ischemic heart disease, Cx43 is lost at the intercalated disc, but Cx43-Ser368 phosphorylation can act to indirectly stabilize the protein [196]. Multiple studies have investigated the effects of targeting the C-terminus of Cx43 in ischemia/reperfusion injuries, reducing infarct size, and other diseases [11,216]. A peptide that mimics the terminal region of the Cx43 known as ACT1 can disrupt Cx43/ZO-1 interaction [214,217]. This peptide promotes phosphorylation of Cx43-Ser368 via upregulation of PKCε activity, inhibits Cx43-ZO-1 binding, and improves cardiac function following ischemic insult in mice [215]. Similar results have been found for other connexin mimetic peptides targeting the Cx43 C-terminus (e.g., antiarrhythmic peptide 10 (AAP10) and rotigaptide (ZP123)), causing increases in Cx43-Ser368 phosphorylation through PKCα (reported to stabilize protein expression and increase GJIC) associated with improved cardiac functions in experimental animal models and early tests demonstrating no adverse effects in humans [192,218,219,220,221,222]. However, it should be noted that a similar peptide, danegaptide (a stabilized form of rotigaptide), failed to change clinical outcomes in ischemic reperfusion injuries in human Phase II testing (NCT01977755, completed 2016) [223].

In vascular disease, phosphorylation-mediated connexin–protein interactions and GJIC have been found to regulate disease state. Oxidized phospholipids found within atherosclerotic plaques increase MAPK and PKC phosphorylation of Cx43 and are associated with increased inflammation and cellular proliferation [130,224,225,226,227]. In response to the release of growth factors in disease, Cx43 is phosphorylated at MAPK residues (Cx43-Ser255, -Ser262, -Ser279, -Ser282), promoting direct interactions with the cyclin E/CDK2 complex and enhancing smooth muscle cell proliferation [131]. Conversely, PKC phosphorylation of Cx37 alters GJIC, which is linked with growth suppressive effects (e.g., reducing vasculogenesis and angiogenesis) [228,229,230,231,232,233]. Mutation of all seven Cx37 Ser > Ala essentially closes Cx37 GJs and hemichannels and inhibits both proliferation and cell death, whereas mutation of only three (Cx37-Ser275, -Ser302 and -Ser328) partially inhibits channel opening and decreases cellular death in rat insulinoma cells [128].

Phosphorylation also plays an important role in altered localization and function of connexins in cancer [3]. Several oncogenes and proto-oncogenes robustly inhibit GJIC, including HRAS [234], c-Src [235], and v-Src [178]. Curiously, the tyrosine-protein kinase c-Src has a reciprocal relationship with Cx43 that regulates its activity, where Cx43 is shown to bind with phosphatases (e.g., PTEN and Csk) reducing c-Src activity [236]. Conversely, Src phosphorylation of tyrosine residues on Cx43 (Cx43-Tyr243/-Tyr265) mediates interactions with endosomal machinery, leading to internalization of Cx43 and reduced expression [118,237]. Numerous tumor promoters, such as phorbol esters, also rapidly inhibit Cx43-mediated GJIC [238,239,240] through PKC- and ERK-mediated phosphorylation events [181,241]. On the contrary, loss of phosphorylation can also negatively affect GJIC. One recent study showed that the levels of total Cx43 protein and Cx43 phosphorylated at Ser368 and Ser279/282 were high in normal tissue but low to absent in malignant pancreatic tissue [79]. Altered Cx43-phosphorylation can be indicative of prognosis in some tumors, such as gliomas [242]. Phosphorylation of other connexins can also affect GJIC and the cancer phenotype, notably PKC-mediated phosphorylation of Cx37 [128]. Targeting dysregulated phosphorylation events of connexins in cancer may be one therapeutic angle towards restoring connexin function or GJIC. Indeed, the chemotherapeutic drug gefitinib has been suggested to upregulate GJIC by inhibiting Src and PKC-modulated Cx43 phosphorylation [243]. However, resistance to cisplatin-based chemotherapy has been suggested to be due to Src-induced Cx43 phosphorylation and loss of GJIC [244].

During wound healing, phosphorylation may also play a role in coordinating GJIC and connexin redistribution [245,246,247]. Initial responses to wounding include a generalized loss in Cx43, which may be modulated by increases in cyclic adenosine monophosphate (cAMP). In wound models, 8-bromo-cAMP-treated embryonic stem cells promote enhanced wound repair associated with reduced membrane bound Cx43, disruption in Cx43-ZO-1 interactions, and reduced GJIC [248]. However, the mechanisms regulating this are unclear, since cAMP-associated kinases have been previously described to increase PKA-mediated Cx43 synthesis, phosphorylation (Cx43-Ser364), GJ assembly, and GJIC in other model systems [189,249]. Phosphorylation is extremely dynamic within the wound and appears to be coordinated with the stage of repair. Initial increases in Cx43-Ser373, driven by AKT, can be seen between 1 and 30 min after wounding occurs, disrupting interactions with ZO-1, initially stabilizing Cx43 at the membrane, but is followed by rapid internalization of Cx43 [200]. Following wounding, transient increases (24–72 h) in PKC-mediated Cx43-Ser368 phosphorylation in regions proximal to the injured sited are associated with a loss of GJIC [250,251]. These data and others suggest that a combination of phosphorylation events sequentially regulate connexin signaling during wound repair [252].

In disease states such as diabetes, nonhealing wounds lead to complications, including ulcerations in skin tissues. In streptozotocin-induced diabetic mice, Cx43 dynamics are different from normal skin tissues, with increased expression of dermal Cx43 associated with reduction in keratinocyte migration [253]. Similar observations have been made in human diabetic ulcers, with Cx43 found to remain at elevated levels as compared to normal skin wounds [254]. In vitro and ex vivo evidence suggests that peptides aimed at disrupting gap junction and hemichannel communication (e.g., Gap27) can improve wound healing, which is associated with increased Cx43-Ser368 phosphorylation [251]. Recent studies have also shown that increases in Cx43-Ser368 phosphorylation following topical application of the ACT1 peptide is associated with clinically significant improvements in scar reduction and wound closure rates [255].

### 3.2. S-Nitrosylation

*S*-Nitrosylation occurs through covalent binding of nitric oxide (NO) to reactive cysteine(s) and can result in structural alterations of proteins leading to functional changes [256]. Protein *S*-nitrosylation is highly dependent on the cysteine oxidation state and surrounding amino acids, meaning that not all cysteines in a protein can be *S*-nitrosylated. While there are cysteine residues on the extracellular loops of all connexins, these have not been demonstrated to be *S*-nitrosylation targets [257]. Within the C-terminus of Cx43, there are three cysteines (Cx43-Cys260, -Cys271, and -Cys298), but only Cx43-Cys271 has been demonstrated to be *S*-nitrosylated, leading to an increase in GJ permeability in endothelial cells and at the MEJ [184]. Direct *S*-nitrosylation of other connexins has not been demonstrated, although there are multiple lines of evidence demonstrating that nitric oxide (NO) activation leads to regulation of gap junction and hemichannel signaling [258]. Within the vasculature, NO plays an important role in vasodilation. Figueroa et al. found that vascular connexins channels formed by Cx37, Cx40, and Cx43 are activated by and directly permeable to NO, and have suggested that this is an alternative method to NO transfer across plasma membranes [259]. Cx37 is enriched at the MEJ of resistance arteries and is reported to be important in the regulation of NO-mediated Ca^2+^ regulation via reducing Cx37-mediated gap junctional coupling between endothelial cells and smooth muscle cells [260]. However, unlike Cx43, the effects of NO on Cx37 gap junction channels are thought to be indirect, with no known cysteine modification occurring. Rather, the phosphorylated tyrosine residue (Cx37-Tyr332) is protected from de-phosphorylation by Src homology region 2 (Shp2) phosphatase, which is inhibited in the presence of NO, reducing MEJ transfer of Ca^2+^ signaling through Cx37 GJ [261]. Thus, *S*-nitrosylation appears to have diverse effects, depending on GJ composition particularly at the MEJ [261].

### 3.3. Other Post-Translational Modifications: SUMOylation, Ubiquitination, and Acetylation

A number of post-translational modifications are associated with regulation of connexin protein turnover, for example, ubiquitination, SUMOylation, and acetylation. Small ubiquitin-like modifier proteins (e.g., SUMO-1/-2/-3) interact with lysine residues on proteins, altering protein targeting and turnover [262]. So far, there is only evidence for direct Cx43 SUMOylation at lysine residues (Cx43-Lys144, -Lys237) within its intracellular loop and C-terminus [172]. Overexpression of all three SUMO proteins in HeLa cells increases Cx43 expression, promotes gap junction formation, and increases signaling. However, the exact mechanism by which SUMOylation regulates protein expression is not known. The amino acids sequences surrounding Cx43-Lys144 and Cx43-Lys237 are not common motifs associated with SUMOylation, although the same motifs of a conserved Lys144 followed by an upstream large hydrophobic amino acid (valine) are found in at least six other connexins, suggesting a common regulatory pathway [172].

Once at the plasma membrane, the majority of connexins are rapidly turned over with half-lives estimated between 1.5 and 5 h for Cx43 and Cx26 and up to 24 h for other isoforms such as Cx46 [124,263,264,265,266]. While connexins use a multitude of pathways for internalization and degradation, the process typically involves formation of an endosome (termed connexosome [267]), where older gap junctions are internalized to be targeted to the lysosome for degradation, although there is also evidence for endosomal recycling back to the membrane [214,268,269]. Endosomal formation is driven by multiple proteins in complex, including interactions with ZO-1, tubulin, and others. In the case of Cx43, this interaction (with ZO-1) is regulated via Cx43-Ser373 and Cx43-Ser368 phosphorylation [200,214,270,271]. Monoubiquitinylation typically acts as a signal for internalization of proteins via endosomes to lysosomes, leading to degradation [272,273]. Multiple covalently linked ubiquitin molecules bind lysine residues within the target protein, which are then recognized by receptors and targeted for degradation by the 26S proteasome [274,275,276] and by autophagy [277,278,279]. Recent evidence has demonstrated a complementary role for Cx43 in regulating autophagy, in that Cx43 at the plasma membrane interacts with several pre-autophagosomal proteins, including Atg16, but not other autophagosome proteins, such as LC3 [280]. When the cells are under stress, such as nutrient depletion, Cx43 becomes ubiquitinylated and internalized, causing recruitment of other factors (Atg5, Atg12, and LC3) to form fully functional autophagosomes. While regulated autophagy can have a protective effect in stressed cells, there is also evidence linking aberrant autophagy and Cx43 degradation from intercalated discs to heart failure [281], suggesting the potential for a novel pharmacologic approach to treat cardiac failure.

Proteasomal–ubiquitin pathways have been proposed to indirectly regulate Cx43 through interaction with the ZO-1 protein, thus disrupting part of the process that is critical for Cx43 membrane organization [214,282]. Multiple studies suggest that other connexin proteins (e.g., Cx50, Cx43, and Cx31.1) are regulated by ubiquitination [283]. Several studies show that ubiquitin regulates internalization of Cx43 via clathrin-mediated endocytosis, by both tyrosine (Y)-dependent sorting signal (YXXΦ, where X is any amino acid and Φ is an amino acid with a bulky hydrophobic side chain) and tyrosine-independent, EPS15-dependent pathways [284,285]. However, the route through which ubiquitin regulates the connexins has not been fully delineated, with studies in Cx43 demonstrating that the C-terminal lysines are dispensable for protein turnover [286]. Despite this, there is increasing evidence that Cx43 is modified in response to ubiquitin, and corresponding ligases are controlled in part by phosphorylation events, such as MAPK and PKC phosphorylation [287,288]. A number of ubiquitin-binding proteins (e.g., EPS15, p62, Hrs, and TSG101) are rec ruited to Cx43 to facilitate its internalization and sorting to the lysosome [289,290]. In addition, TSG101 has been found to interact with Cx30.2, Cx31, Cx36, and Cx45 [290]. While classic lysine-based motifs may not be responsible for direct ubiquitin binding, more recent studies have shown that proline-rich regions of the Cx43 C-terminus (xPPxY) bind to ubiquitin ligase. A number of ubiquitin ligases have also been associated with direct binding, internalization, and degradation of Cx43 (e.g., Trim21 [291], WWP1 [292], SMURF2 [293], and NEDD4 [287,288,289,294]). NEDD4 also has been directly associated with loss of Cx43 at the plasma membrane in experimental models [287].

The process of degradation may be further regulated by connexin N-terminal acetylation, which can act to regulate protein stability in the membrane. In mouse cardiac myocytes, N-terminal acetylation through binding of P300/CBP-associated factor with Cx43 leads to a loss of Cx43 at the intercalated disc, a lateral reorganization of the protein, reduced gap junction formation in cardiac myocytes, and internalization in NIH-3T3 (mouse embryo) fibroblasts [77]. These patterns of disorganization of Cx43 are similar to those seen in mouse models of Duchenne cardiomyopathies, where NO and oxidative stress lead to an imbalance in acetylation/deacetylation and alterations in cardiac conduction. Similarly in dogs, cardiac pacing leads to increased Cx43 acetylation, suggesting that this mechanism is important in regulating signaling in physiology and pathology of the cardiac system [77,295,296].

## 4. Connexin Trafficking

Formation of gap junctions by connexins is regulated by the delivery of newly synthesized channels to the plasma membrane and is balanced by the removal of channels via endocytosis [263,297,298]. As mentioned above, since connexin turnover is generally quite rapid and influenced by post-translational modifications, the dynamic regulation of connexins by secretion and turnover provides a means to control gap junction formation, composition, and, thus, GJIC.

### 4.1. Control of Oligomerization

Secretion of newly synthesized connexins from the endoplasmic reticulum (ER) through the Golgi apparatus is coordinately regulated with oligomerization into hexameric hemichannels [299]. Based on structural homology, connexins can be separated into two distinct oligomerization groups. *GJB1*–*GJB7* (so-called β connexins, including Cx26 and Cx32) follow a more traditional pathway, where full oligomerization into hexamers is required prior to transport from the ER to the cis-Golgi apparatus [300,301,302]. By contrast, other connexins are stabilized by a connexin-specific quality control apparatus as monomers that are subsequently transported to the trans-Golgi network (TGN) where they then have the capacity to oligomerize [301,303]. The best-studied connexin known to oligomerize in the TGN is Cx43, although there is also experimental evidence for Cx40 and Cx46 oligomerization late in the secretory pathway [304,305]. By homology, it is likely that most non-beta connexins will also follow the late oligomerization pathway that has been demonstrated for Cx43 [299].

**Figure 2:**
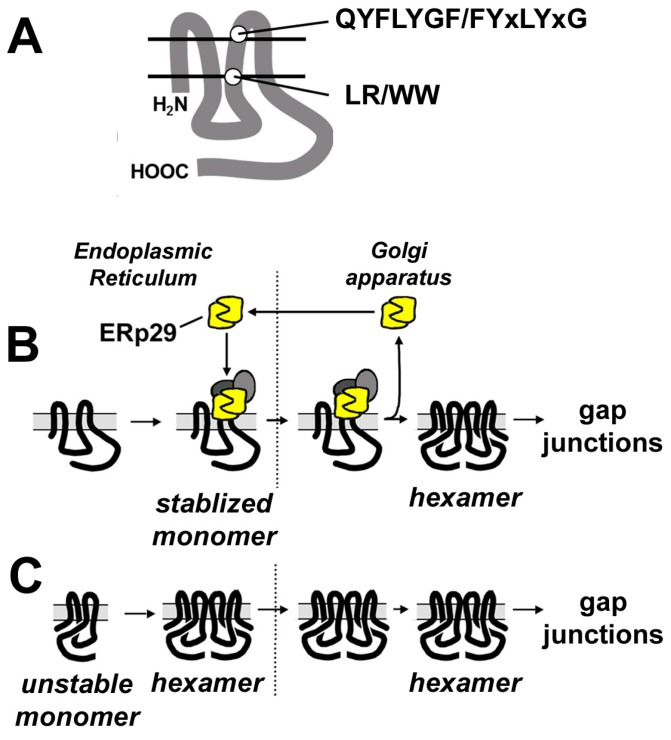
Differential connexin oligomerization. (**A**) Line diagram showing two key connexin motifs adjacent to the third transmembrane domain. Connexins (such as Cx43), which oligomerize in the Golgi apparatus (**B**), have a cytosolic LR and extracellular QYFLYGF motif that interacts with ERp29 (yellow) and other putative chaperones (grey ovals) that stabilize monomeric connexins until they transition from the endoplasmic reticulum (ER) to the Golgi apparatus (delineated by the dashed lines). In the Golgi apparatus, ERp29 dissociates from monomeric connexins and then recycles back to the ER, enabling connexins to oligomerize into hexameric hemichannels. By contrast, connexins (such as Cx32) that have a WW and a FYxLYxG motif cannot interact with ERp29—they are inserted into the ER membrane as unstable monomers and thus immediately oligomerize (**C**).

Several lines of evidence suggest that the transition from monomeric to hexameric Cx43 requires a conformational change, largely centered on the third transmembrane domain (TM3) where it is stabilized in a monomeric conformation by motifs containing charged amino acids on both ends of the TM domain (Figure 2) [300,305]. At the cytoplasmic interface of the Cx43 TM3 domain is an LR motif containing a highly charged arginine residue, and at the extracellular interface is a glutamine-containing motif with a QYFLYGF amino acid consensus sequence. The extracellular loop domain of Cx43 also interacts with a chaperone protein, ERp29, that is required to stabilize monomeric Cx43 [300].

By contrast, beta connexins lack charged residues adjacent to the TM3 domain. They instead have a di-tryptophan (WW) motif that is less stringently localized to the membrane/cytosol interface, and they lack the ability to interact with ERp29. Thus, beta connexins are not stable as monomers and instead oligomerize in the ER (Figure 2) [300,301,302]. Since motifs associated with the TM3 domain also have been implicated in regulating connexin hetero-oligomerization [299,306], this implicates a role for spatial separation of connexin oligomerization in regulating the extent and stoichiometry of heteromeric channel formation.

### 4.2. Connexin Quality Control

The differences in quality control for Cx26 and Cx43 were directly observed for native connexins in human airway epithelial cells derived from a cystic fibrosis (CF) patient expressing the CF transmembrane conductance regulator (CFTR) protein harboring the Fdel508 mutation [307]. In these cells, Cx43 trafficking and function is impaired, yet Cx26 transport and assembly into gap junction channels is normal. Interestingly, CFTR also interacts with ERp29 [308], and Cx43-mediated GJIC by Fdel508-CFTR-expressing cells is restored by treatment with 4-phenylbutyrate, a drug that upregulates ERp29 expression [307,308]. In addition, 4-phenylbutyrate has been shown to upregulate GJIC in several other contexts [309,310,311,312,313,314], further underscoring a role for ERp29 and other 4-phenylbutyrate-sensitive factors in connexin quality control.

Aberrant accumulation of connexins in the ER clearly decreases the pool of connexins available to produce gap junction channels at the cell surface. However, ER accumulation of connexins has also been found to induce an unfolded protein response (UPR) that, in turn, has the capacity to impair cell function and lead to human disease. UPR induced by mutant connexins has been directly demonstrated for Cx50 mutations associated with cataract [315,316,317] and Cx31 mutations that cause the skin disease erythrokeratoderma variabilis (EKV) [318] or hearing impairment [319]. The association of UPR with human diseases related to misfolded connexins suggests the possibility that treatments alleviating ER stress, such as 4-phenylbutyrate, may have therapeutic value by promoting proper protein folding and trafficking as well as increasing GJIC. Also, as mentioned above, the ability of 4-phenylbutyrate to enhance GJIC also may contribute to its potential as an anticancer therapeutic, and may be related to increased ERp29 activity [320].

### 4.3. Connexin Cytoplasmic Domains and the Cytoskeleton

In addition to motifs adjacent to the TM3 domain, there are several lines of evidence in support of connexin C-terminal domains in regulating connexin trafficking. As described above, in addition to containing several motifs that can be post-translationally modified, the semi-structured nature of the C-terminus [153,155,321] enables it to be conformationally labile and to interact with several different classes of cytosolic scaffold proteins and the cytoskeleton that can influence connexin targeting (reviewed in [5] for Cx43). For instance, several truncated connexins lack the ability to be efficiently trafficked to the plasma membrane or be endocytosed [322,323]. The connexin C-terminal domains also have the capacity to homo- and hetero-dimerize [153,155,159,324] as well as interact with other connexin domains, including the cytoplasmic loop [155,325,326] that can influence connexin targeting, oligomerization, and function.

Interestingly, it was determined that there is reciprocal regulation of Cx43 and Cx46 in the lens, where conditions such as activation of PKC caused an increase in Cx46 transcription and expression that was associated with a concomitant decrease in Cx43, via ubiquitination and proteasomal degradation [327]. In fact, transfecting cells with Cx46 was sufficient to induce Cx43 degradation and this effect required the C-terminus of Cx46, since a Cx46 tail truncation mutant had no effect on Cx43 expression. Increased Cx50 also had no effect on Cx43. However, transfecting cells with a soluble Cx46 tail construct had the ability to decrease Cx43 expression. Since the decrease in Cx43 was induced by an intracellular pool of Cx46, this raises the possibility that crosstalk between Cx46 and Cx43 may be related to differential oligomerization [304]. However, this remains to be determined.

As another instance where the C-terminus plays a key role in regulating Cx43 trafficking, it has been shown that amino N-terminal truncated forms of Cx43 are also expressed by cells, through alternative internal translation via one of six different AUG initiation sites (see Section 2.4.2) [328]. The most prominent of these is GJA1-20k, which consists of a portion of the TM4 domain as well as the entire C-terminus [110] (Figure 1). GJA1-20k expression promotes formation of Cx43 gap junction channels, resulting in an increase in intercellular communication [110,113]. As discussed in Section 2.4.2, alternative translation of Cx43, including production of GJA1-20k, is inhibited by mTOR [110,111] and Mnk1/2 kinases [111], suggesting that metabolic stress regulates gap junctional coupling through mTOR- and Mnk1/2-mediated pathways as a means to protect cells both by enabling scarce metabolites to be distributed via intercellular communication as well as limiting damage by restricting generation of reactive oxygen species [329].

How GJA1-20k regulates channel formation by Cx43 is still under investigation. One intriguing possibility is that GJA1-20k acts as a chaperone protein that promotes Cx43 oligomerization, as was recently demonstrated to regulate the decrease in gap junction formation and function that can occur in the epithelial to mesenchyme transition [330] (Figure 1).

Another likely role for GJA1-20k relates to cytoskeletal control of Cx43 trafficking, since it has been shown that the C-terminus of Cx43 and, therefore, GJA-20k as well, interacts with both microtubules and filamentous actin [331,332,333]. Microtubules and actin perform complementary functions in regulating connexin trafficking, where microtubules help facilitate rapid transport of Cx43-containing vesicles to sites of junction formation [332], whereas actin has a more subtle role in regulating connexin trafficking, since quantitative live cell-imaging shows that transport of Cx43-containing vesicles temporarily pauses when they interact with actin filaments, perhaps as a means to enhance sorting or to remodel vesicle composition [331]. Also, transfecting HeLa cells with GJA1-20k nucleates the formation of actin filaments [113], suggesting a role for GJA1-20k in altering the itinerary of Cx43 trafficking in the cell. Reverse regulation is also suggested by studies where gap junction inhibitors resulted in misalignment of actin filaments across the monolayer and reduced calcium signaling in rat astrocytes [334]. Furthermore, treatment of astrocytes with an actin polymerization inhibitor cytochalasin D or anti-actin antibodies reduced GJIC, as visualized by a reduction in the spread of microinjected neurobiotin between cells [335].

### 4.4. Regulation of Gap Junction Plaque Morphology

Actin has also been implicated in regulating gap junction plaque morphology. Double knockout of the actin capping protein tropomodulin 1 and intermediate filament protein CP49 in lens fiber cells led to a significant decrease in Cx46 plaque volume and increase in plaque number, affecting gap junction coupling and function in the lens tissue [336]. Regulation of plaque size by actin is likely to be coordinated by interactions involving the C-terminus of connexins and zonula occludens 1 (ZO-1). For example, enhanced green fluorescent protein (EGFP)-tagged Cx43 incapable of interacting with ZO-1 produces plaques that are not size regulated [337]. By contrast, the perimeter of gap junction plaques (the perinexus) is ringed by Cx43/ZO-1 complexes, whereas the center of plaques is largely devoid of ZO-1 [338]. Inhibition of Cx43/ZO-1 interactions cause an increase in gap junction plaque size. Consistent with this possibility, Cx43 phosphorylation inhibits ZO-1 binding and facilitates connexin channel endocytosis [339]. Additional roles for ZO-1, connexin phosphorylation, and ubiquitinylation in regulating connexin endocytosis and degradation are described in Section 3.2 and Section 3.3, above.

Although the precise mechanism whereby ZO-1 limits plaque formation is still under investigation, it seems plausible that it may be analogous to the role of ZO-1 in regulating tight junctions, where claudin/ZO-1/actin interactions have a junction-stabilizing influence on the apical junctional complex, whereas, in the absence of ZO-1, there is increased access of myosin that increases tight junction dynamics and tension [340,341]. Consistent with this possibility, myosin VI has also been found to have a specific role in increasing gap junction plaque size, analogous to treatments inhibiting Cx43/ZO-1 interactions [342].

Whether regulation of plaque assembly strictly follows the perinexus model has recently been challenged by observations of Cx36 plaque formation [343]. Pulse-chase experiments with Cx36 indicated addition of Cx36 to both the ends and the middle of preexisting gap junction plaques, with diffusion of Cx36 throughout the plaque. When the experiments were repeated with Cx43, there appeared to be less diffusion of newly added Cx43 in preexisting plaques [343]. Targeted delivery of connexins has only recently been observed. Through interactions with plus-end binding protein EB1 and the dynein/dynactin complex, microtubule plus-ends are tethered to adherens junctions at the plasma membrane, leading to the targeted deposition of connexin hemichannels and gap junction plaque formation [332]. These two models begin to bring to light the vast complexity of connexin trafficking and gap junction formation, suggesting a network of cytoskeleton and protein-binding partners tailored to specific connexins that was previously unrealized.

A less understood but intriguing role for the cytoskeleton in gap junction biology is the creation of unique junctional subregions involved in gap junction dynamics. Using an EGFP-tagged Cx32 construct, particularly dynamic regions at the edges of gap junction plaques were observed as invaginated tubular structures, where plaque fragments pinched off into the cytoplasm [344]. These tubulovesicular extensions of gap junction plaques were recently observed with Cx36 and termed filadendrites [345]. Filadendrites at the edges of gap junction plaques appeared to be the same thickness as the plaque, suggesting that the filadendrites were a continuation of the gap junction plaques themselves. Filadendrites were also observed in interior regions of the gap junction plaques, but appeared to be much thinner than the gap junction plaques. From pulse-chase labeling of Cx36, it was observed that filadendrites exhibited some of the same dynamic properties as the earlier observed Cx32 invaginations, constantly pinching off and fusing with the gap junction plaque. Labeling of actin filaments showed colocalization with Cx36 filadendrites, suggesting that the actin cytoskeleton could be one of the drivers behind the formation of these dynamic structures. Treatment with the actin polymerization inhibitor Latrunculin A or actin depolymerization inducer cytochalasin D reduced the presence of filadendrites, indicating that the driving force behind the dynamic gap junction plaques requires actin polymerization [345].

Similar structures have been noted at other junctions. Primary human keratinocytes treated with pemphigus vulgaris (PV) IgG containing antibodies targeted to the adherens junction protein desmoglein 3 (Dsg3) exhibited reorganized Dsg3 at the membrane into projections perpendicular to the membrane plane. These projections, termed linear arrays, are similar to the filadendrites in that they are sites of disassembly of junction components and active endocytosis at the junctions. Linear arrays also colocalized with actin filaments oriented perpendicular to the plasma membrane, similar to those observed in filadendrites. Furthermore, linear arrays were associated with decreased cell adhesion, suggesting a functional effect of these junctional subregions [346,347]. A comparable structure formed by tight junction proteins, termed tight junction spikes, have been observed to correlate with treatments that enhance junction disassembly and paracellular leak, including oxidative stress induced by chronic alcohol exposure, transforming growth factor (TGF)-β1 treatment, and inhibition of NF-κB [348,349,350]. In alveolar epithelial cells, actin filaments colocalized with the tight junction protein claudin-18 in tight junction spikes. Spikes were also found to be sites of budding and fusion of vesicles carrying tight junction proteins, both indicators of active tight junction remodeling. Treatment of lung alveolar epithelial cells with granulocyte-macrophage colony-stimulating factor (GM-CSF) reduced actin filament colocalization with claudin-18 containing tight junction spikes, whereas keratinocyte growth factor treatment inhibited spike formation and instead promoted formation of cortical actin as opposed to actin stress fibers [350,351]. Taken together, these findings indicate that these similar junctional subregions observed universally across several different classes of intercellular junctions, including gap junctions, could represent a common mechanism of junction protein turnover, where the junctions partition themselves into unique filamentous structures. Whether these structures serve to restrict turnover of junction proteins to specific subdomains or whether they nucleate the formation of signaling complexes that recruit specialized subsets of cytosolic binding partners remains to be determined.

## 5. Conclusions and Future Perspectives

In order to fully understand the complex role of connexins in health and disease, it is essential to elucidate their regulation at all steps, from gene transcription, protein synthesis, post-translational modifications, and trafficking to their regulation at the cell membrane. This review is intended to highlight some of the progress made in these areas in relation to health and disease, giving examples of how this knowledge is pertinent for future therapeutic applications. Going forward, understanding how modulation of connexins occurs at any of these stages will require additional work and insight, which over time may lead to more fruitful and safer strategies to alleviate patient suffering. For example, the danegaptide trials that were based on strong preclinical data suggested that alterations to the trafficking and increased Cx43 signaling in the heart would have a profound effect in reducing ischemic reperfusion injury and reduce cardiac tissue damage. However, Phase II clinical trials in humans failed to show an effect, highlighting the complex nature of targeting gap junctions as a treatment modality including differences in how connexins are regulated in model systems as opposed to human disease. Additional caution is also needed for therapeutic approaches in cancer, where it is now clear that connexins have distinct roles that both promote and inhibit cell growth and metastasis.

Despite substantial progress, it is important to acknowledge the complexity of gap junctions that serve as a conduit that enables cells to share thousands of different signaling molecules. Additionally, the complex connexin protein interactome underscores the non-junctional functions of connexins, including their ability to act as a signaling platform. In particular, it is critical to identify connexin-specific functions that are unique and targetable. This is best approached by understanding how connexins are regulated at multiple levels by multiple mechanisms, from gene transcription and translation to post-translational modification, and as a specifically localized multiprotein complex.

## Figures and Tables

**Table 1:** Connexin post-translational modifications (PTMs) and functional effects.

Connexin/Residue	PTM	GJIC	Expression	Refs.
**Cx26 ^m.s./a^:**				
M1/K15/K102/ K103/K105/ K108/K112/K116	Acetylation	ND	ND	[138,139]
N14/N113/ N170/N176	Hydroxylation	ND	ND	
E42/E47/E114	carboxylation	ND	ND	
K61/R75/ K221/K223	Methylation	ND	ND	
T123/T177/S183/ T186/(Y233 or Y235 or Y240)	Phosphorylation	ND	ND	
**Cx31(m):**				
263 ^b^	CK1	No change	No change	[124]
266 ^b^	CK1	No change	No change	[124]
**Cx32:**				
S229	PKC	Increase/Decrease	Increase/Decrease	[165]
S233	PKA/PKC	Increase/Decrease	Increase/Decrease	[120,138,165,166]
S240	ND	ND	ND	[138]
Y7/Y243	EGFR tyrosine kinase	ND	ND	[167]
**Cx35/Cx36:**				
S110	PKA/PKG	No change	Decrease	[141,168,169,170]
S276/293	PKA/PKG	No change	Decrease	[141,168,169,170,171]
S289	PKG (NO mediated)	ND	Decrease	[170]
**Cx37:**				
S275/S285/ S302/S319/S321/ S325/S329	PKC	Increased	Decrease	[128]
**Cx43:**				
S5 ^m.s.^	ND	ND	ND	[144]
K144	SUMO	Increase	Increase	[172]
K237	SUMO	Increase	Increase	[172]
S244 ^m.s.^	CAMKII	ND	ND	[173]
Y247 ^c^	Src	Decrease	Decrease ^c^	[120,146,174,175,176,177,178]
S255 ^m.s.^	CAMKII	ND	ND	[173]
	P34cdc2	Decrease	Decrease	[179,180]
	MAPK	No change/Decrease	No change	[120,131,148,181]
S257 ^m.s.^	PKG/CAMKII	ND	ND	[173]
S262 ^d^	P34cdc2	Decrease	Decrease	[179,180]
	MAPK	Decrease	Decrease/no change	[120,131,148,182,183]
	PKCε ^a^	Decrease	Decrease	[131,181,182]
Y265 ^c^	Src	Decrease	Decrease ^c^	[120,146,174,175,176,177,178]
C271	Nitrosylation	Increase	No change	[184]
S279 ^e^	MAPK	Decrease	Decrease/no change	[131,148,174]
	CDK5		Decrease	[185]
S282 ^e^	MAPK	Decrease	Decrease/no change	[131,148,174]
	CDK5	Decrease	Decrease	[185]
S296 ^m.s.^	CAMKII	ND	No change	[173,186]
S297 ^m.s.^	CAMKII/PKCε	ND	No change	[173,186]
S306 ^m.s.^	CAMKII	Decrease	Decrease associated with De-Phosph.	[173,186,187]
S314 ^m.s.^	CAMKII	ND	ND	[173]
S325 ^m.s.^	CAMKII	ND	ND	[173]
	CK1	Increase	Increase	[188]
S328 ^m.s.^	CAMKII	ND	ND	[173]
	CK1	Increase	Increase	[188]
S330 ^m.s.^	CAMKII	ND	ND	[173]
	CK1	Increase	Increase	[188]
S364 ^m.s.^	CAMKII	ND	ND	[173]
	PKA	Increase	Increase	[120,189,190,191]
S365 ^m.s.^	CAMKII	ND	ND	[173]
	PKA	Increase	Increase	
	PKC	Decrease	Decrease	[120,192,193,194]
S368 ^f^	PKCα	Increase/Preserved/	Increase	[192,193,194,195,196]
	PKCε	Decrease ^g^	Decrease	[120,193,194,195,196,197,198,199]
S369 ^m.s.^	CAMKII	ND	ND	
	PKA	Increase	No change	[173]
	PKC	Increase	Increase	[5,120,192,193,194]
S372 ^m.s.^	CAMKII	ND	ND	[173]
	PKC	Decrease	Decrease	[5,148,192,193,200,201]
S373 ^g,m.s.^	Akt	Increase	Increase ^e^	[200,201]
	CAMKII	ND	ND	[173]
	PKC	Decrease	Decrease	
	PKA	Increase	Increase	[120,148,192,193,194]
**Cx45:**				
S326/Y337/ S381/S382/S384/ S385/S387/S393 ^m.s.^	CAMKII	ND	ND	[129]
S326/S382/S384/ S387/S393 ^m.s.^	CK1	ND	ND	[129]
**Cx46 (Cx56 Chick homologue):**				
S118	PKCε	ND	Decrease	[140,202]
**Cx50:**				
S363	CK1	Increase	Increase	[120,193]

Notes: ^a^ mass spec identified a number of potential phosphorylation sites in Cx26 but did not test functions, although mutations at many of these sites are associated with disease pathology [139]. ^b^ Direct phosphorylation not shown, S263 and S266 on Cx31 contain consensus sequence for Ck1 which, when deleted, alters functions. ^c^ Src may not alter function of formed gap junctions. ^d^ Currently debated as to whether Cx43-S262 is a CDK1/CDC2/PKC/MAPK site, and several lines of evidence indicate that this is most likely an ERK-regulated site [148]. ^e^ Functions of S279/S282 typically shown by single phosphorylation antibodies or multiple site directed mutagenesis including both residues. Decrease GJIC as a result of reduced open probability. ^f^ Phosphorylation of S368 by phorbyl esters, e.g., TPA, are associated with PKCε phosphorylation and reduced communication. In ischemia, treatment by peptides, e.g., rotagaptide, increase S368 phosphorylation by PKCα, leading to increases in GJIC. ^g^ While initial phosphorylation at S373 is associated with a temporal increase in GJ size, it is thought to be the start in the process that leads to internalization. Abbreviations: ND, not demonstrated; (m), mouse; m.s., mass spectrometry-based identification approach. GJIC, gap junction intercellular communication; EGFR, epidermal growth factor receptor; PKA/PKC, Protein kinase A/ Protein kinase C; NO, nitric oxide; SUMO, small ubiquitin-like modifier.
